# Effect of H_2_S Plasma Treatment on the Surface Modification of a Polyethylene Terephthalate Surface

**DOI:** 10.3390/ma9020095

**Published:** 2016-02-05

**Authors:** Alenka Vesel, Janez Kovac, Gregor Primc, Ita Junkar, Miran Mozetic

**Affiliations:** Department of surface engineering, Jozef Stefan Institute, Jamova 39, Ljubljana 1000, Slovenia; janez.kovac@ijs.si (J.K.); gregor.primc@ijs.si (G.P.); ita.junkar@ijs.si (I.J.); miran.mozetic@guest.arnes.si (M.M.)

**Keywords:** hydrogen sulfide (H_2_S), plasma treatment, polymer, polyethylene terephthalate (PET), surface modification

## Abstract

H_2_S plasma created by an electrode-less radio-frequency discharge was used to modify the surface properties of the polymer polyethylene terephthalate. X-ray photoelectron spectroscopy, secondary ion mass spectrometry and atomic force microscopy were used to determine the evolution of the surface functionalities and morphology. A very thin film of chemically bonded sulfur formed on the surface within the first 10 s of treatment, whereas treatment for more than 20 s caused deposition of higher quantities of unbonded sulfur. The sulfur concentration reached a maximum of between 40 and 80 s of plasma treatment; at longer treatment times, the unbonded sulfur vanished, indicating instability of the deposited sulfur layer. Large differences in the surface morphology were observed.

## 1. Introduction

Plasma created in various gases are often used for altering the surface properties of polymer materials. Oxygen and nitrogen-containing plasmas (O_2_, CO_2_, N_2_, NH_3_) or their mixtures with noble gases, such as He and Ar, are commonly used for surface hydrophilization [1,2,3,4,5]. In contrast, fluorine-containing plasmas (CF_4_, SF_6_) are used for surface hydrophobization [6,7]. Recently, a few papers have reported on tailoring the surface properties by SO_2_ plasma [8,9,10]. SO_2_ plasma could be useful for polymer modification in biomedical applications, e.g., preparation of antithrombogenic surfaces or for altering cell adhesion. However, there are almost no reports in the scientific literature regarding the use of other sulfur-containing plasmas, such as H_2_S, for polymer modification.

An interesting application of gaseous plasma created in H_2_S is the decomposition of this hazardous gas into hydrogen and sulfur. Such decomposition has attracted serious attention recently because it enables the production of hydrogen from a hazardous H_2_S waste gas, which is a byproduct of oil refinement [11,12,13,14,15,16,17]. Currently, the conventional treatment method for H_2_S destruction is the Claus process, which produces sulfur, whereas hydrogen is converted to water and therefore lost [12,18]. This is the reason why plasma has been investigated as a promising alternative technique for H_2_S decomposition into hydrogen and sulfur and thus for hydrogen production, which is of strong commercial interest [19,20,21]. 

Another possible application of H_2_S plasma is the synthesis of thiol (-SH) groups on the polymer surfaces upon treatment in H_2_S plasma. Such -SH functionalities can serve as anchoring sites for the immobilization of macromolecules in biosensors and disease diagnostics, or they can act as a support for gold nanoparticles [22,23,24,25]. Classical methods for the synthesis of thiol groups are based on wet chemistry procedures that use different solvents, require long reaction times and depend on substrate surface properties [22]. Therefore, dry, solvent-free processes, like gaseous plasmas, are a good alternative to classical methods. Thiry *et al.* synthesized a plasma polymer film containing -SH groups [22,23,24,25]. He did not use H_2_S plasma but performed plasma polymerization using propanethiol (CH_3_CH_2_CH_2_SH) as a precursor. Furthermore, other authors reported the formation of sulfur-rich surfaces by plasma polymerization using thiophene as a precursor [26,27,28,29,30].

The literature survey indicates interesting applications of H_2_S plasma in various fields, from oil chemistry to biopolymers; therefore, it is worth investigating the mechanisms of the interaction with solid materials. In this paper, we report observations on the surface modification of polyethylene terephthalate (PET) polymer treated in H_2_S plasma.

## 2. Results and Discussion

### 2.1. Plasma Characterization

**Figure 1:**
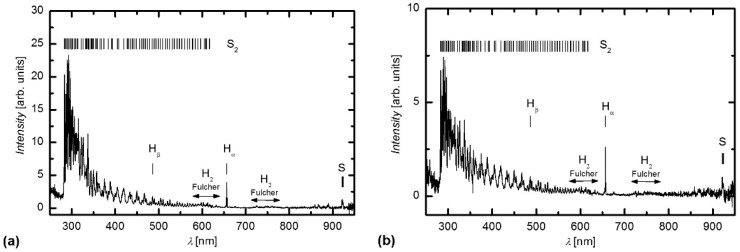
OES spectra of H_2_S plasma (**a**) without; and with (**b**) PET samples.

Figure 1a shows an optical emission spectrum (OES) of radiofrequency (RF) H_2_S plasma created at a pressure of 30 Pa and RF power of 150 W. The spectrum is rich in S_2_ molecular transitions. Only a few atomic lines are observed, and they correspond to H_α_, H_β_ and S. The transitions at approximately 600 nm and 750 nm correspond to the H_2_ Fulcher band [31], whereas the transitions between 280 and 620 nm correspond to S_2_ molecular transitions [32]. The S_2_ emission band B-X from 283 to 306 nm is particularly strong. Figure 1b shows the OES spectrum of plasma during sample treatment. The samples were placed in the middle of the RF coil. The spectrum is similar to the spectrum obtained for the empty tube.

### 2.2. Surface Chemistry

**Figure 2:**
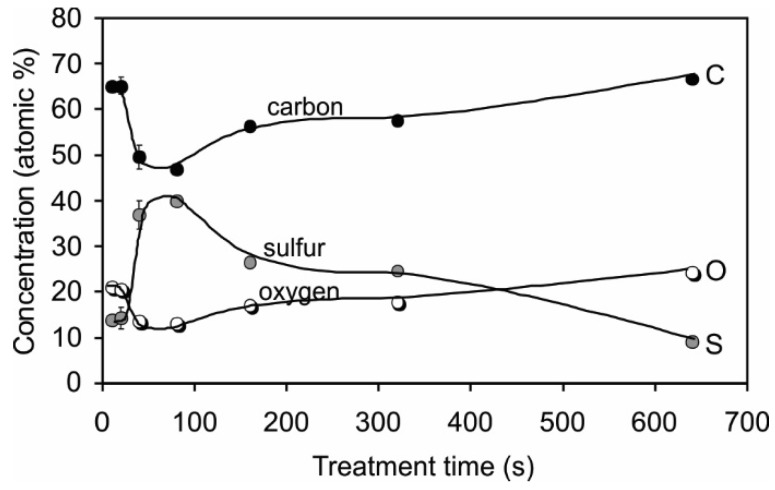
XPS surface composition of PET samples treated in H_2_S plasma for various periods.

Figure 2 shows the surface composition of PET samples treated in H_2_S plasma for various periods as deduced from the XPS (X-ray photoelectron spectroscopy) survey spectra. The samples were not rinsed before analysis to remove loose material but were rather quickly transferred to an XPS chamber to minimize aging effects. Plasma treatment resulted in the appearance of a significant sulfur content at the surface. The sulfur content was not constant, and it depended on the treatment time. At lower treatment times, the sulfur content was approximately 14 atomic %. At 40 s of treatment, the sulfur content increased significantly, and it reached a maximum of 40 atomic % at 80 s of treatment. At longer treatment times, the sulfur concentration slowly decreased, and at 640 s of treatment, the content was only 9 atomic %. The opposite variation of the concentration was observed for carbon and oxygen, *i.e.*, the minimum concentration of carbon and oxygen was observed at the maximum sulfur concentration.

**Figure 3:**
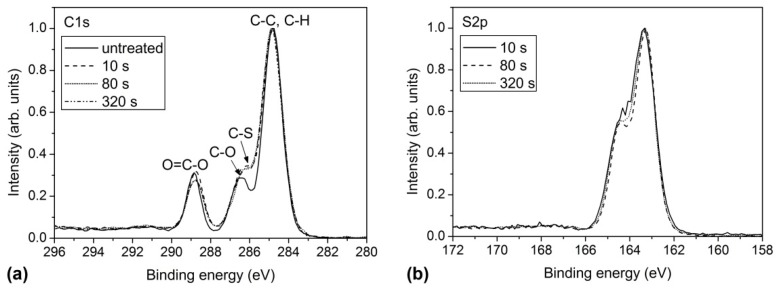
Comparison of the high-resolution XPS spectra of the (**a**) carbon C1s peak; and (**b**) the sulfur S2p peak at various treatment periods.

To explain the unusual behavior of the sulfur concentration, we recorded high-resolution XPS spectra to obtain additional information regarding the surface chemical composition. Figure 3 shows the high-resolution XPS spectra of the carbon and sulfur peaks for the selected treatment periods. When comparing the carbon spectra of various plasma-treated samples, we did not find a significant difference in the shape of the spectra. The only difference was observed when comparing the spectra of plasma-treated samples with the untreated sample. The local minimum at 286 eV between the C-C and C-O peaks was less pronounced, which was due to the appearance of a new sub-peak corresponding to the C-S bonds at approximately 285.5 eV [33].

Further information was obtained from Figure 3b, which shows the comparison of the high-resolution sulfur peaks. Sulfur peaks for plasma-treated samples were positioned at approximately 163.3 eV, indicating that sulfur was bonded either to another sulfur atom or to carbon. The absence of peaks at higher binding energies close to 170 eV indicated that sulfur was not bonded to oxygen atoms in PET. According to the results shown in Figure 3, sulfur was bonded to carbon atoms in -CSH or similar groups (C-S-S, C=S, C-S-S). Unfortunately, the chemical shifts corresponding to different sulfur functional groups are too small to allow for reliable conclusions about the surface chemistry involved. Nevertheless, the maximum sulfur concentration on the surface was so high that it could not be explained solely by surface functionalization with sulfur-containing functional groups. The XPS technique gives the concentration of elements averaged over the investigation depth, which is estimated to several nm. 

**Figure 4:**
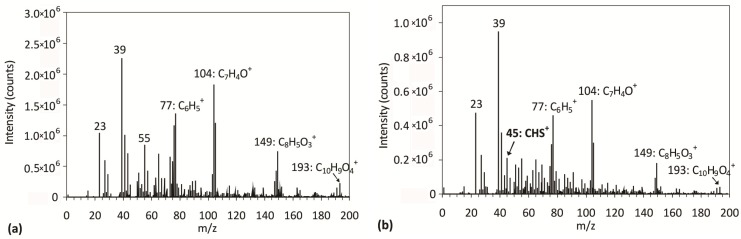
Positive SIMS spectra for (**a**) untreated PET; and (**b**) PET treated for 80 s.

**Figure 5:**
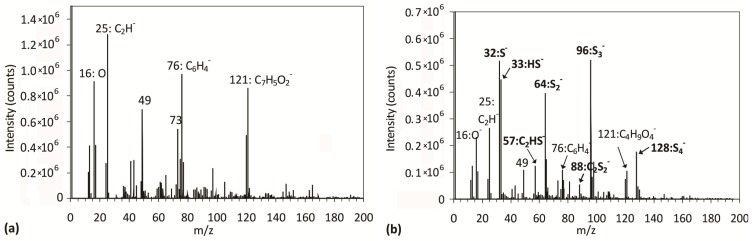
Negative SIMS spectra for (**a**) untreated PET; and (**b**) PET treated for 80 s.

To obtain more information regarding the chemical changes at the plasma-treated surface, we performed SIMS (secondary ion mass spectrometry) measurements. Positive and negative SIMS spectra for an untreated sample and a plasma-treated sample, where the maximum sulfur content was observed by XPS, are shown in Figure 4 and Figure 5, respectively.

The positive SIMS spectrum for an untreated sample (Figure 4a) reveals characteristic peaks at *m*/*z* 77 (C_6_H_5_^+^), 104 (C_7_H_4_O^+^), 149 (C_8_H_5_O_3_^+^) and 193 (C_10_H_9_O_4_^+^), which correspond to characteristic molecular fragments of PET polymers [34]. In the negative SIMS spectrum of the untreated sample (Figure 5a), we observe the following major characteristic PET fragments: m/z 76 (C_6_H_4_^−^) and 121 (C_7_H_5_O_2_^−^). For the plasma-treated sample (the positive SIMS spectrum is shown in Figure 4b), we can see that all characteristic PET peaks remain in the spectrum. Furthermore, a new peak at *m*/*z* = 45, assigned to CHS^+^, appeared as a consequence of plasma treatment. Additional information for the plasma-treated sample can be found in the negative SIMS spectrum shown in Figure 5b, where we can see characteristic peaks arising from S_4_^−^ (*m*/*z* 128), S_3_^−^ (*m*/*z* 96), S_2_^−^ (*m*/*z* 64) and S^−^ (*m*/*z* 32) molecules, which indicates that the sulfur accumulates on the polymer surface. Other peaks can be found as well: HS^−^ (*m*/*z* 33), C_2_HS^−^ (*m*/*z* 57) and S_2_C_2_^−^ (*m*/*z* 88).

**Figure 6:**
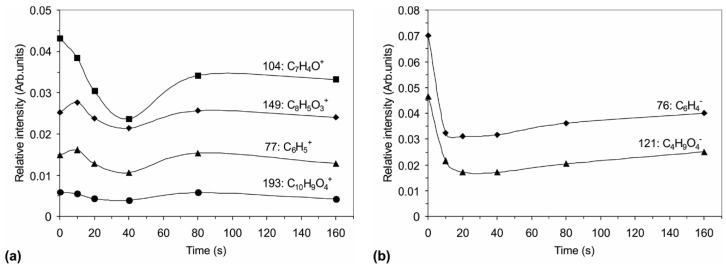
Variation of the relative SIMS intensities of PET fragmented ions: (**a**) positive; and (**b**) negative.

**Figure 7:**
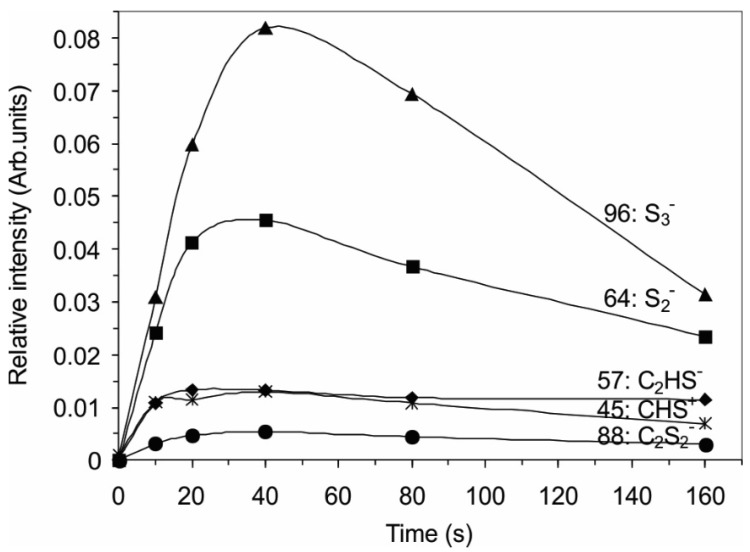
Variation of the relative SIMS intensities of the positive and negative fragments, which are linked with sulfur.

Figure 6 and Figure 7 show the variation of the relative intensities of the main positive and negative SIMS signals with treatment time. The diagrams in Figure 6 show the intensities of the fragments correlated with the PET polymer, whereas Figure 7 shows the fragments containing sulfur atoms. The intensity of the characteristic PET positive signals (Figure 6a) at first decrease with increasing treatment time, reaching a minimum, whereas, at longer treatment times, the original intensities are almost restored. A similar conclusion can be drawn for the variation of the negative signals of the characteristic PET fragments with treatment time (Figure 6b). However, for fragments containing sulfur, we observe the opposite variation with treatment time (Figure 7)—there is a distinctive maximum, similar to the one observed in the case of XPS measurements.

By comparison of the results obtained by XPS and SIMS, we can explain such a high concentration of sulfur on the polymer surface by its accumulation rather than chemical bonding to carbon atoms from the polymer. The surface-reaction mechanism is thus as follows: the initial step in the interaction of plasma with the polymer is chemical binding of sulfur to carbon atoms. This conclusion can be drawn from the behavior of C_2_HS^−^, CHS^+^ and C_2_S_2_^−^ in Figure 7. The surface quickly saturates with sulfur, forming a thin film of chemically bonded sulfur. The necessary treatment time for saturation of the surface with sulfur atoms chemically bonded to carbon can be estimated from Figure 7 as almost 20 s. Once the concentration of chemically bonded sulfur reaches an almost constant value (treatment time over 20 s), simultaneously with chemical bonding, the sulfur atoms also accumulate on the surface of the treated polymer. The deposition of sulfur is revealed from the appearance of the S_2_^−^ and S_3_^−^ peaks in the negative SIMS spectra. The intensity of these spectral features increases strongly for the first 40 s of plasma treatment and then decreases slowly, as revealed from Figure 7. This layer of sulfur, which is not chemically bonded to carbon of the polymer substrate, may be in the form of polysulfides -S_x_- because sulfur tends to catenate (bind to itself by the formation of chains), but the formation of any type of polysulfide is almost impossible to confirm by our experimental techniques. The appearance of unbonded sulfur has been described in several reports, in which plasma-assisted decomposition of H_2_S was investigated to develop a method for destroying this environmentally problematic gas, which is produced in the oil refinement industry [13,17,18,19,20]. These studies found direct decomposition into H_2_ and S, which was deposited on the reactor wall [19]. A similar deposition on the walls of the plasma reactor was observed in our study, as revealed from Figure 7.

**Figure 8:**
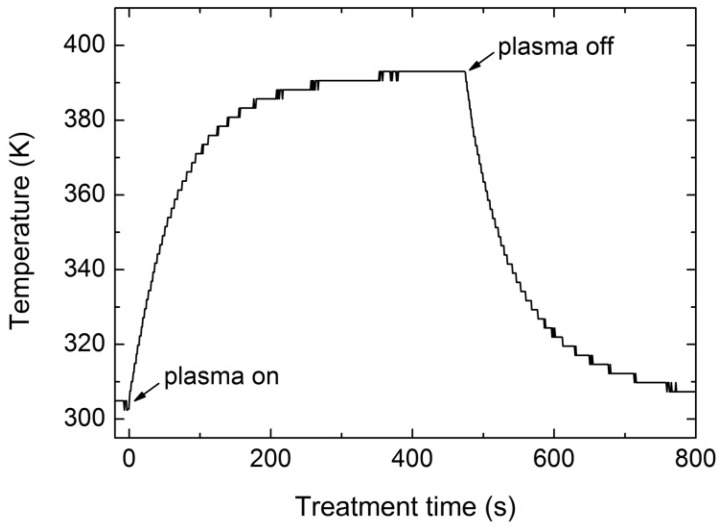
Sample temperature during plasma treatment.

It was unexpected that, at longer treatment time, the quantity of sulfur at the surface was significantly reduced. The deposited sulfur layer must be unstable in our experimental conditions because both the XPS and SIMS results clearly demonstrated slow but continuous decreasing of the S concentration for prolonged treatment periods. The layer formed upon the first minute of plasma treatment must have been removed from the surface upon prolonged treatment. The reason for this may be degradation as a result of the higher surface temperature because polysulfides (R-S_x_), as well as other sulfur compounds, such as polysulfanes (H_2_S_x_), are not thermally stable [35,36]. Furthermore, the process of degradation may be accelerated by UV radiation from plasma [35]. To estimate the possible thermal effect, we measured the sample temperature during plasma treatment. The result is shown in Figure 8. This figure shows that the bulk temperature of the sample reached more than 90 °C at 200 s of treatment, which is enough to cause degradation of thermally unstable compounds and desorption to the gas phase. Our results presented in Figure 2, Figure 6 and Figure 7 indicate that even a somewhat lower temperature is high enough to facilitate desorption of sulfur compounds under low-pressure conditions. Thiry *et al.* observed a similar effect: at treatment conditions where the surface temperature was low (30 °C–35 °C), the sulfur concentration was much higher than at conditions where the surface temperature was moderate (60 °C–90 °C) [25]. He explained this effect as being caused by trapped H_2_S molecules in the surface film, which were released at high temperatures.

### 2.3. Surface Morphology, Etching and Aging

**Figure 9:**
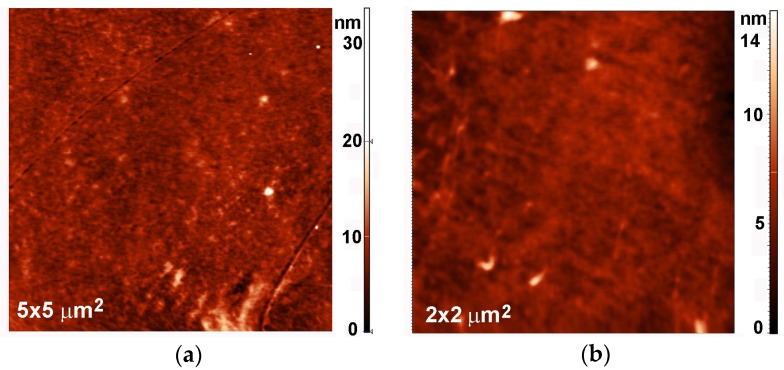
AFM images of untreated PET sample: (**a**) 5 × 5 μm^2^; and (**b**) 2 × 2 μm^2^.

**Figure 10:**
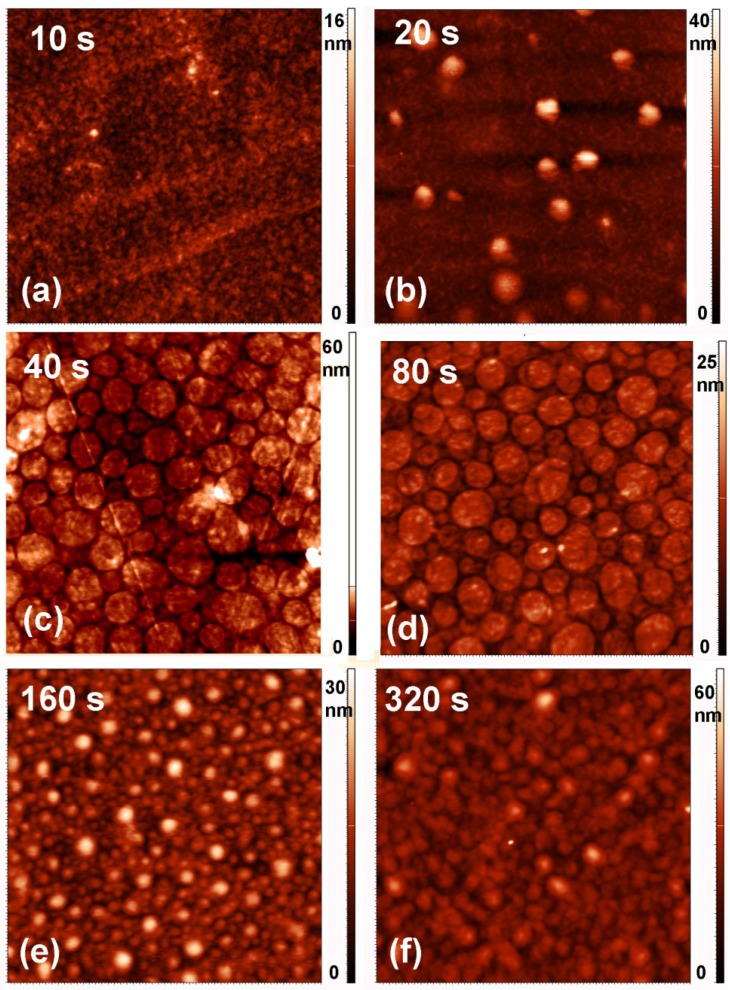
AFM images (5 × 5 μm^2^) of samples treated for various periods.

**Figure 11:**
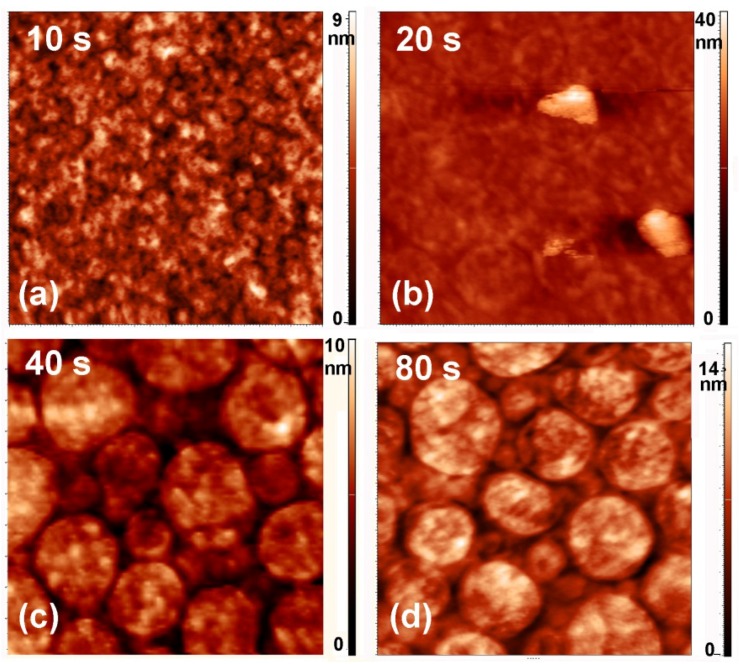

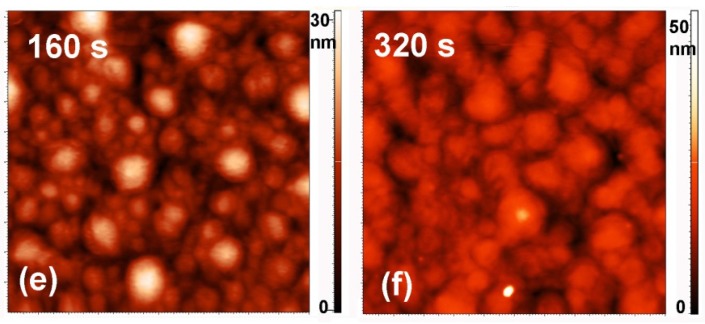
AFM images (2 × 2 μm^2^) of samples treated for various periods.

Any deposition of a foreign material on the substrate may or may not be reflected in modified surface morphology, depending on the growth mechanisms. To investigate the morphological changes, we performed AFM (atomic force microscopy) analyses. Figure 9 shows an AFM image of an untreated PET sample, whereas Figure 10 and Figure 11 show AFM images of selected samples recorded on 5 × 5 μm^2^ and 2 × 2 μm^2^ areas, respectively. The corresponding surface roughness is shown in Table 1. The roughness evolution deduced from the AFM images roughly coincides with the behavior of the sulfur concentration determined by XPS and SIMS. Initially (up to the treatment time of 10 s), the surface is quite smooth without any special features (Figure 10a and Figure 11a). At 20 s of treatment, the formation of the first particles at the surface is initiated (Figure 10b and Figure 11b). At 40 s of treatment, the surface is fully covered with particles, and their lateral size and height have increased (Figure 10c and Figure 11c). These particles can be attributed to large sulfur clusters on the surface, but as shown later in the text, this conclusion does not agree with other observations. After 80 s of treatment, the surface roughness has decreased; however, the surface is still fully covered with circular features (Figure 10d and Figure 11d). At longer treatment periods, when the sulfur concentration measured by XPS and SIMS has decreased, the lateral size of the particles has also decreased (Figure 10e,f and Figure 11e,f).

**Table 1:** Surface roughness of the samples treated for different periods as measured by AFM over an area of 2 × 2 µm^2^ and 5 × 5 µm^2^.

Treatment Time (s)	Roughness (nm)	
	Measured on 5 × 5 µm^2^ Area	Measured on 2 × 2 µm^2^ Area
0	1.2	1.2
10	1.2	1.2
20	2.4	1.6
40	1.7	1.4
80	2.3	2.1
160	3.3	3.1
320	3.8	3.2

**Figure 12:**
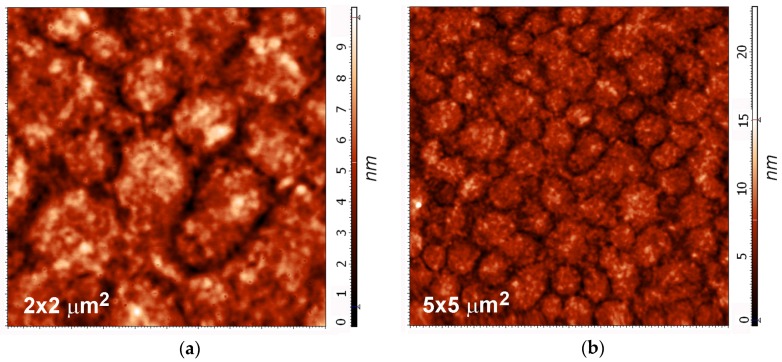
AFM images (**a**) 2 × 2 μm^2^; and (**b**) 5 × 5 μm^2^ of the PET sample treated for 80 s after six months of aging.

Even though the AFM results show a good correlation with the XPS, and especially the SIMS, the clusters observed in the AFM images are not associated with the sulfur deposit. The sample with the maximum sulfur concentration was aged in air for six months and then analyzed again (Table 2). The XPS results of the aged sample showed a significant decrease in the sulfur concentration. After half a year of aging, the concentration was only approximately 6 atomic % (initially, it was 40 atomic %). The AFM images showed that the surface morphology remained unchanged, and clusters are still observed (Figure 12).

**Table 2:** Surface composition of the sample with the maximum sulfur concentration (treated for 80 s) after aging for different periods (in atomic %).

Aging Period	Element Concentration (atomic %)		
	Carbon	Oxygen	Sulfur
0 days	46.9	13.1	40.0
1 day	65.0	18.5	16.4
2 days	67.2	19.1	13.7
6 months	73.8	19.8	6.4

Therefore, the observed surface morphology cannot be explained by sulfur deposition or by intensive etching of the polymer because no oxygen lines were observed in the OES spectra (Figure 1b). Furthermore, the etching rates of polymer exposed to hydrogen radicals and ultraviolet (UV) or vacuum ultraviolet (VUV) radiation are small [37], but they are unknown for SH radicals. Therefore, we performed weight loss measurements to estimate the etching rate. Selected samples were first weighed, treated in plasma and rinsed with toluene to remove sulfur deposits, and then weighed again. Knowing the surface area, sample density and treatment time, we calculated the etching rate, which was approximately 1.5 nm/s. Although the etching rate was small, it caused changes to the surface morphology. The etching was highly non-uniform due to sulfur deposition. Deposition of sulfur caused certain areas of the surface to be covered with sulfur and were therefore protected, whereas the other parts of the surface were exposed to etching. At longer treatment periods, when sulfur was removed, the surface was etched more uniformly, and the surface morphology was changed again.

### 2.4. Surface Wettability

**Figure 13:**
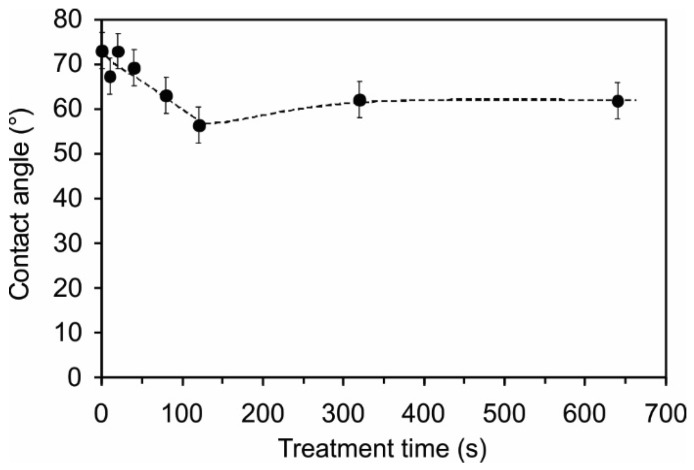
Water contact angle variation *vs.* plasma treatment period.

Because the surface roughness and morphology in combination with the surface functionalization may have a significant effect on the surface wettability, we also measured the water contact angles on the plasma-treated samples. The contact angles are shown in Figure 13. For untreated PET, the angle was approximately 73°, whereas for plasma-treated samples, the angle was between 57° and 72°. Comparing the contact angles with the XPS, SIMS and AFM results, it is difficult to find any correlation. Therefore, we can only conclude that the moderate hydrophobic nature of this polymer is preserved upon treatment with H_2_S plasma.

## 3. Materials and Methods

### 3.1. Plasma Treatment

**Figure 14:**
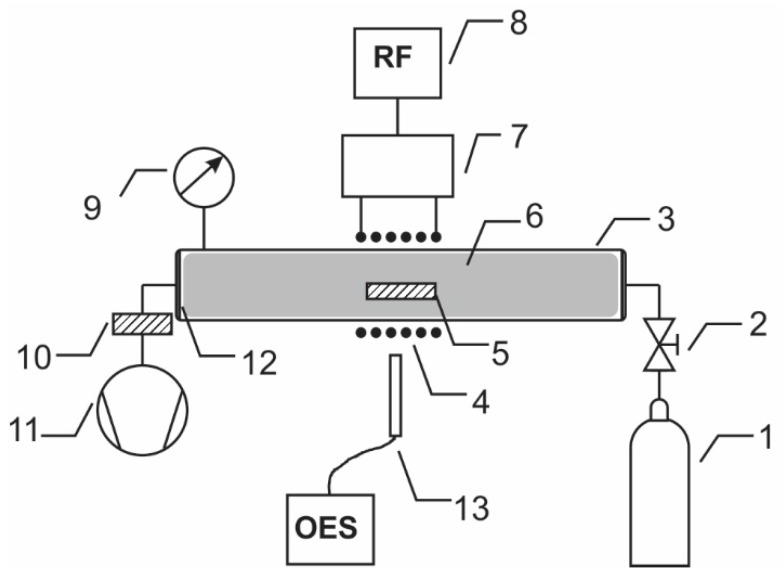
Schematic diagram of the experimental system: 1—H_2_S gas source; 2—leak valve; 3—discharge tube; 4—coil; 5—sample; 6—plasma; 7—matching network; 8—RF generator; 9—vacuum gauge; 10—catalyzer; 11—vacuum pump; 12—flange; 13—OES spectrometer.

Biaxially oriented polymer PET from Goodfellow Ltd. (Huntingdon, UK) was used. The polymer film with a thickness of 0.125 mm was cut to small square pieces with a size of 1 × 1 cm^2^. The samples were treated in the discharge tube presented schematically in Figure 14. The tube was made from Pyrex glass and was 80 cm long and 4 cm in diameter. The discharge tube was pumped with a rotary pump operating at a nominal pumping speed of 80 m^3^·h^−1^. Hydrogen sulfide (H_2_S) gas was leaked into the experimental system on the opposite side, as shown in Figure 14. The pressure was set to 30 Pa, so the gas flow rate was 400 sccm. A coil of 6 turns was mounted in the center of the Pyrex tube, as shown in Figure 14. Plasma was created by an RF generator coupled to the coil via a matching network. The matching network consisted of two vacuum capacitors: one connected in series and another in parallel. The generator operated at the standard frequency of 13.56 MHz, and its nominal power was set to 150 W. At these discharge conditions, the plasma was ignited in the low-density mode (*i.e.*, E-mode) [38]. Samples were treated in H_2_S plasma for various periods of 10, 20, 40, 80, 160, 320 and 640 s. After treatment, the samples were characterized using atomic force microscopy (AFM), X-ray photoelectron spectroscopy (XPS), time-of-flight-secondary ion mass spectrometry (ToF-SIMS), and contact angle measurements. Some samples were stored and re-characterized after prolonged time to monitor ageing effects.

### 3.2. Plasma Characterization

Plasma was characterized using optical emission spectroscopy (OES). OES measurements were performed in a quartz tube with a 16-bit Avantes AvaSpec 3648 fiber optic spectrometer (Avantes Ltd., Leatherhead, Surrey, UK). The nominal spectral resolution was 0.8 nm, and spectra were recorded in the range from 200 to 1100 nm. A combined deuterium tungsten reference light source was used to determine the spectral response of the spectrometer. The measured OES spectra were calibrated with this spectral response. Because the light emitted by H_2_S plasma at our discharge parameters was weak, the integration time used to record the OES spectra was 10 s.

### 3.3. Temperature Measurements

Temperature of the sample during plasma treatment was measured by a chromel-alumel thermocouple. The thermocouple tip was placed between two pieces of PET film, which were pressed together to make good contact with the thermocouple.

### 3.4. Weight-Loss Measurements

The etching of the polymer during plasma treatment was monitored by measuring the weight loss of the selected polymer samples. The samples were weighed just before mounting into the plasma reactor and again just after plasma treatment. A Radwag XA 110 (Radwag, Radom, Poland) professional microbalance was used. The accuracy of the measurements was, according to the producer, 0.01 mg Samples were washed in toluene and dried before weighing to remove any impurities, deposits or degradation products from the surface.

### 3.5. AFM Measurements

An AFM (Solver PRO, NT-MDT, Moscow, Russia) was used to characterize the topology of the samples. All measurements were performed in tapping mode using ATEC-NC-20 tips (Nano And More GmbH, Wetzlar, Germany) with a resonance frequency of 210–490 kHz and force constant of 12–110 Nm^−1^. The surface roughness was calculated from the AFM images taken over an area of 2 × 2 µm^2^ and 5 × 5 µm^2^ using the program Spip 5.1.3 (Image Metrology A/S, Hørsholm, Denmark). The surface roughness was expressed in terms of the average roughness (Ra).

### 3.6. XPS Measurements

XPS characterization of the polymer samples was performed to determine their chemical composition after plasma treatment using an XPS (TFA XPS Physical Electronics, Münich, Germany). The samples were excited with monochromatic Al K_α1,2_ radiation at 1486.6 eV over an area with a diameter of 400 µm. Photoelectrons were detected with a hemispherical analyzer positioned at an angle of 45° with respect to the normal of the sample surface. XPS survey spectra were measured at a pass-energy of 187 eV using an energy step of 0.4 eV, and high-resolution spectra were measured at a pass-energy of 23.5 eV using an energy step of 0.1 eV. An additional electron gun was used for surface neutralization during the XPS measurements. All spectra were referenced to the main C1s peak of the carbon atoms, which was assigned a value of 284.8 eV. The measured spectra were analyzed using MultiPak v8.1c software (Ulvac-Phi Inc., Kanagawa, Japan, 2006) from Physical Electronics, which was supplied with the spectrometer.

### 3.7. ToF-SIMS Measurements

ToF-SIMS analyses were performed using a ToF-SIMS 5 instrument (ION-TOF, Münster, Germany) equipped with a bismuth liquid metal ion gun with a kinetic energy of 30 keV. The analyses were performed in an ultra-high vacuum of approximately 10^−7^ Pa. The SIMS spectra were measured by scanning a Bi_3_^+^ cluster ion beam with a diameter of 1 μm over a 100 × 100 μm^2^ analysis area. The positive secondary ion mass spectra were calibrated using CH_2_^+^, CH_3_^+^, and C_2_H_5_^+^, and the negative secondary ion mass spectra were calibrated using C^−^, C_2_^−^, and C_3_^−^. An electron gun was used for charge compensation on the sample surfaces during the analysis.

### 3.8. Contact Angle Measurements

The surface wettability was measured immediately after plasma treatment by determining the water contact angle (WCA) with a demineralized 3 μL water droplet. Contact angles were measured by See System (Advex Instruments, Brno, Czech Republic). For each sample, five measurements were taken to minimize the statistical error. The contact angles were determined by the software supplied by the producer.

## 4. Conclusions

PET polymer was modified with radicals created in the gaseous plasma of H_2_S. Plasma was sustained by an electrode-less RF discharge created in the E-mode, which enabled ionization and dissociation of H_2_S molecules to form H and HS radicals. Combination of different surface-sensitive techniques allowed for insight into the surface chemistry as well as morphological changes upon plasma treatment. A couple of competitive processes were identified: (i) chemical bonding of sulfur atoms to carbon on the substrate surface; and (ii) deposition of a thin sulfur film. The first process was irreversible because the concentration of the sulfur bonded to carbon atoms remained unchanged even after prolonged aging. The thin sulfur film, however, was unstable. The concentration of the chemically unbonded sulfur decreased with prolonged plasma treatment time and almost vanished after storing for several months.

The concentration of total sulfur on the polymer surface increased with treatment time for the first minute, which was explained by the accumulation of sulfur on the surface. This layer is thermally unstable and degrades spontaneously, even at room temperature. Slow decrease of the sulfur concentration for prolonged plasma treatment times (over a minute) was observed and was attributed to the increasing polymer temperature. After several minutes of plasma treatment, the sulfur concentration significantly decreased and reached a few atomic % after approximately 10 min. The results indicate the existence of an optimal treatment time to obtain a large concentration of sulfur. At our experimental conditions, the optimal time is approximately one minute, but this value is different under other conditions due to the different heating of the polymer samples. Large changes of the surface morphology (formation of spherical features) were observed, but were not directly related to sulfur deposition.
